# Two male siblings with a novel *LRBA* mutation presenting with different findings of IPEX syndrome

**DOI:** 10.1099/jmmcr.0.005167

**Published:** 2018-10-15

**Authors:** Sanem Eren Akarcan, Neslihan Edeer Karaca, Guzide Aksu, Ayca Aykut, Deniz Yilmaz Karapinar, Funda Cetin, Yesim Aydinok, Elif Azarsiz, Eleonora Gambineri, Ozgur Cogulu, Ezgi Ulusoy Severcan, Hudaver Alper, Necil Kutukculer

**Affiliations:** ^1^​ Department of Pediatric Immunology, Ege University Faculty of Medicine, Izmir, Turkey; ^2^​ Department of Medical Genetics, Ege University Faculty of Medicine, Izmir, Turkey; ^3^​ Department of Pediatric Hematology, Ege University Faculty of Medicine, Izmir, Turkey; ^4^​ Department of Pediatric Gastroenterology, Ege University Faculty of Medicine, Izmir, Turkey; ^5^​ Department of Hematology-Oncology: Bone Marrow Transplant (BMT) Unit, University of Florence, Department of ‘‘NEUROFARBA’’: Section of Child’s Health, ‘‘Anna Meyer’’ Children’s Hospital, Florence, Italy; ^6^​ Department of Pediatric Radiology, Ege University Faculty of Medicine, Izmir, Turkey

**Keywords:** LRBA deficiency, IPEX phenotype, lung infections, enteropathy, autoimmunity, abatacept

## Abstract

**Introduction:**

LPS-responsive beige-like anchor (LRBA) protein deficiency is a disease of immune dysregulation with autoimmunity affecting various systems.

**Case Presentation:**

Two male siblings with a novel *LRBA* mutation had different primary findings at admission: the younger sibling had chronic early-onset diarrhoea and the elder one had autoimmune haemolytic anaemia. During long-term follow-up for IPEX phenotype, both developed hypogammaglobulinaemia, enteropathy and lung involvement. The patients partially responded to immunosuppressive therapies. A homozygous c.2496C>A, p.Cys832Ter (p.C832*) mutation in the *LRBA* gene causing a premature stop codon was detected. After molecular diagnosis, abatacept, as a target-specific molecule, was used with promising results.

**Conclusion:**

LRBA deficiency is a recently defined defect, with variable presentations in different patients; a single, definitive treatment option is thus not yet available.

## Introduction

The immune system has distinct and interrelated cellular and humoral components composed of many cells, signalling pathways, cytokines and receptors. There are 354 primary human immune deficiency diseases with 344 gene defects, grouped into nine different categories with respect to common phenotypes [1].

LPS-responsive beige-like anchor protein (LRBA) deficiency is a relatively newly defined molecular defect, first described in 2012, in five patients with severe, early-onset hypogammaglobulinaemia and autoimmune manifestations. Susceptibility to inflammatory bowel disease (IBD), and recurrent infections were important characteristics of these patients [2]. In a similar time frame, another investigator group found *LRBA* mutation in five members of a large family presenting with IBD. Autoimmune cytopenia, renal disease, recurrent infections and lymphoproliferative disease were other findings [3]. LRBA deficiency was first classified in common variable immune deficiency disorders, but as the identified cases increased and different cohorts of *LRBA* mutant patients were published, the spectrum of the phenotype has expanded [4–6]. LRBA deficiency is now categorized as a disease of immune dysregulation with autoimmunity [1].

LRBA is a cytosolic protein which controls cytotoxic T lymphocyte-associated protein-4 (CTLA-4) expression [7]. CTLA-4 is a protein found in endocytic vesicles and released to the cell surface of regulatory T cells (Tregs) with T cell receptor (TCR) stimulation. It is an inhibitory molecule competing with the co-stimulatory molecule CD28 for the same ligands (CD80 and CD86) on antigen-presenting cells [8]. The amount of CTLA-4 at the cell surface is important for inhibition of self-reacting T cells after antigen recognition. LRBA binds to the cytosolic tail of CTLA-4 and blocks its transport to lysosomes for removal [7]. Both heterozygous *CTLA-4* and biallelic *LRBA* mutations result in insufficient functional CTLA-4, abolishing CTLA-4-dependent Treg function and causing immune dysregulation and autoimmunity [9].

Herein, we present two male siblings with a novel homozygous *LRBA* mutation with both similar and different clinical features (Table 1), expanding the possible diagnoses until the exact molecular defect was defined. These siblings were followed for immune dysregulation, polyendocrinopathy, enteropathy, X-linked (IPEX) or IPEX-like syndrome for several years.

**Table 1.** Clinical findings of the two patients

	Patient 1	Patient 2
Current age (years)	10	11
Age at first symptoms (years)	Diarrhoea: 1	Recurrent bronchiolitis: 1 Haemolytic anaemia: 5.5
Age at admission (years)	3	7
Failure to thrive	(+)	(−) at admission Developed during follow up
Recurrent respiratory infections	(+)	(+)
Hepatosplenomegaly	(+)	(+)
Lymphadenopathy	(+)	(+)
Lung involvement	Bronchiectasis Granulomatous lung disease (radiology diagnosis)	Lymphocytic interstitial pneumonia (pathology diagnosis)
Endocrinological involvement	Compensatory hypothyroidism Antithyroid antibody-positive	(−)
Gastrointestinal involvement	Duodenitis, colitis	Duodenitis, microscopic colitis
Kidney involvement	Chronic glomerulonephritis	Focal segmental glomerulosclerosis
Autoimmune cytopenias	(−)	Haemolytic anaemia Thrombocytopenia Neutropenia

## Case report

### Patient 1

A 3-year-old boy was admitted to inpatient clinics with complaints of persistent diarrhoea and failure to thrive. He had watery, mucous, sometimes bloody defecation 3–4 times a day since 1 year of age. He was born via vaginal delivery at week 32 of gestation with birth weight of 2200 g. He had been investigated for chronic diarrhoea in a public hospital before admission to our department. Cystic fibrosis was excluded with negative sequence analysis. He had low complement 3 (C3) and C4 values with proteinuria. Kidney biopsy revealed minimal interstitial fibrosis. He was the second child of second-degree consanguineous healthy parents with three live births. The first male child and third female child were known to be healthy at that time. His paternal uncle had died in his second decade due to IgA nephropathy and chronic renal failure.

He showed failure to thrive (weight −2.03 standard deviation score (SDS), height −1.89 SDS, under the third percentile), multiple dental caries, coarse lung sounds and a 1/6 systolic murmur on his first admission. He had iron deficiency anaemia [haemoglobin (Hb): 8.5 g dl^−1^, mean corpuscular volume (MCV): 66 fl, iron: 16 µg dl^−1^, total iron binding capacity (TIBC): 268 µg dl^−1^, transferrin saturation: 5.9 %, ferritin: 7.9 g l^−1^), hypoproteinaemia and hypoalbuminaemia (total protein: 5.8 g dl^−1^, albumin: 2.9 g dl^−1^). C-reactive protein was 0.7 mg dl^−1^ (normal: <0.3 mg dl^−1^), and erythrocyte sedimentation rate was 40 mm h^−1^ (normal: <20 mm h^−1^). Stool examinations for lipid, blood, parasites and viruses were negative, but *Salmonella enteritidis* grew in stool culture. Thyroid hormones were within normal limits, except a mild thyroid-stimulating hormone (TSH) elevation [TSH: 6.4 µIU ml^−1^ (normal: 0.35–5.5)] and he had been followed for compensatory hypothyroidism before admission. He had non-nephrotic proteinuria [14 mg m^–2 ^h^–1^ (normal: <4 mg m^–2 ^h^–1^)]. Abdominal ultrasonography (US) was normal.

He had normal IgG and IgM levels, a complete IgA deficiency and low IgE level. C3 and C4 were under the lower limit of normal values (Table 2). Lymphocyte subgroups revealed a low percentage of B cells (CD19+ cells: 4.4 %) (Table 2). Specific antibody response for *Hemophilus influenza* was sufficient, whereas tetanus was absent. He had positive cytomegalovirus (CMV) DNA in serum (481 copies ml^−1^ by PCR) without clinical findings. Autoantibodies (antinuclear antibody, antithyroid peroxidase, antithyroglobulin antibodies) were negative while direct Coombs test was positive.

**Table 2.** Serum immunoglobulin, complement levels, lymphocyte subgroups as percentages and absolute cell numbers compared to age-related reference values (at admission and during follow-up) [25–27]

	Patient 1	Reference values		Reference values		Reference values	Patient 2	Reference values		Reference values
	Age 3	Mean±sd (Min–Max)	Age 5	Mean±sd (Min–Max)	Age 10	Mean±sd (Min–Max)	Age 7	Mean±sd (Min–Max)	Age 11	Mean±sd (Min–Max)
IgG (mg dl^−1^)	664	879.9±157.2 (539–1200)	228	986.2±209.6 (528–1490)	292	1062.8±238.8 (646–1620)	1020	1040.7±203.2 (527–1590)	302	1051.7±228.9 (579–1610)
IgA (mg dl^−1^)	<6.5	68.8±22.2 (40.7–115.0)	<6.5	91.9±37.4 (23.0–205.1)	<6.5	116.7±45.9 (54.0–268.0)	32.3	108.4±42.3 (36.1–268.0)	<6.5	115.8±43.0 (27.0–198.0)
IgM (mg dl^−1^)	77	86.1±35.3 (26.1–188.0)	853	105.8±40.8 (33.3–207.0)	17.3	93.9±49.3 (33.7–257.0)	226	97.6±42.9 (30.5–220.0)	769	102.4±38.8 (30.0–187.0)
IgE (IU ml^−1^)	0.13	2–199			<0.01	2–696	<0.01	2–403		
C3 (mg dl^−1^)*	90.5	90–180	86	90–180	192	90–180	93.8	90–180	109	90–180
C4 (mg dl^−1^)*	7.2	10–40	6.8	10–40	38.6	10–40	6.58	10–40	19.9	10–40
White blood cells (mm^−3^)	6150	9851±2772 (5460–18300)	12 800	9851±2772 (5460–18300)	15 950	7263±2424 (3690–13300)	12 700	7263±2424 (3690–13300)	5340	7263±2424 (3690–13300)
Absolute lymphocyte count (cells mm^−3^)	3200	4555±1517 (1050–9030)	2110	4555±1517 (1050–9030)	2210	2779±921 (1340–5170)	1380	2779±921 (1340–5170)	1120	2779±921 (1340–5170)
CD3+ T cells (%)	87	70.0±7.18 (48.2–81.4)	90	70.0±7.18 (48.2–81.4)	92	71.6±9.51 (48.6–89.4)	76	71.6±9.51 (48.6–89.4)	91	71.6±9.51 (48.6–89.4)
(mm^−3^)	2784	3220±1180 (506–7267)	1900	3220±1180 (506–7267)	2033	1989±709 (804–3837)	1048	1989±709 (804–3837)	1019	1989±709 (804–3837)
CD19+ B cells (%)	4.4	16.5±5.70 (6.7–30.4)	0.8	16.5±5.70 (6.7–30.4)	0.3	13.3±4.78 (4.98–26.3)	21	13.3±4.78 (4.98–26.3)	3	13.3±4.78 (4.98–26.3)
(mm^−3^)	141	739±329 (242–1459)	17	739±329 (242–1459)	7	377±202 (89.1–1067)	290	377±202 (89.1–1067)	34	377±202 (89.1–1067)
CD3+CD4+ Th cells (%)	59	40.3±7.27 (23.2–59.5)	57	40.3±7.27 (23.2–59.5)	30	40.0±10.1 (25–62.9)	37	40.0±10.1 (25–62.9)	20	40.0±10.1 (25–62.9)
(mm^−3^)	1888	1314±542 (118–3245)	1202	1314±542 (118–3245)	663	818±395 (202–1899)	510	818±395 (202–1899)	224	818±395 (202–1899)
CD3+CD8+ Tc cells (%)	24	24.2±5.48 (15.2–39)	31	24.2±5.48 (15.2–39)	60	25.7±5.58 (15.3–40.9)	33	25.7±5.58 (15.3–40.9)	65	25.7±5.58 (15.3–40.9)
(mm^−3^)	768	803±417 (108-236)	654	803±417 (108-236)	1326	515±244 (212–1389)	455	515±244 (212–1389)	728	515±244 (212–1389)
CD3-CD1656+ NK cells (%)	4	11.2±4.85 (3.4–26.4)	5	11.2±4.85 (3.4–26.4)	5	10.0±5.63 (1.47–24.8)	1.4	10.0±5.63 (1.47–24.8)	3	10.0±5.63 (1.47–24.8)
(mm^−3^)	128	509±295 (143–1599)	106	509±295 (143–1599)	110	264±161 (50–721)	19	264±161 (50–721)	34	264±161 (50–721)
CD3+HLA-DR+ active T cells (%)	23	7.84±3.7 (2.1–16.2)	25	7.84±3.7 2.1–16.2	37	8.17±5.0 1.34–20.2	12.4	8.17±5.0 1.34–20.2	32	8.17±5.0 1.34–20.2
(mm^−3^)	736	375±235 (22–954)	528	375±235 22–954	818	241±185 34–680	171	241±185 34–680	358	241±185 34–680
CD3+CD4 CD8-TCRαβ+ T cells (%)			0.39				1.7		0.7	
CD4+CD25+Foxp3+ (%)			1.38		0.75		0.72		0.34	
T-cell proliferation response to mitogens					Normal					Partially reduced

*Complement 3 (C3) and C4 levels were measured with commercially available nephelometric kits (Siemens).

Endoscopic evaluation of the upper and lower gastrointestinal tract showed oesophagitis, nodularity and granulation of the duodenum, granulation and fragility of the caecum, and bleeding foci and microabscesses in the colon. Microscopy revealed intraepithelial increased eosinophilic infiltration and plasma cell infiltration in lamina propria of duodenum, irregular crypt structure, and increased eosinophilic and lymphocytic infiltration in lamina propria of colon linked to allergic/eosinophilic gastrointestinal disorder. Sulfasalazine and corticosteroids were prescribed for enteropathy.

He was discharged with preliminary diagnoses of IPEX syndrome, combined immunodeficiency and common variable immunodeficiency (CVID) with positive pathological findings of autoimmune enteropathy and nephropathy. During follow-up, enteropathy continued with decreasing IgG and increasing IgM levels suggesting immunoglobulin class switch recombination defects. CD40 molecule was positive in 98 % of B cells and CD40L on T cells showed the expected increase upon activation. Regular intravenous immunoglobulin (IVIG) replacement was started.

At the age of 5 years, he was hospitalized with pneumonia. He had growth retardation (weight −2.73 SDS, under 3rd percentile; height −1.21 SDS, 3–10 percentile), submandibular, cervical, axillary lymphadenomegalies reaching 2 cm, crackles in lungs and hepatosplenomegaly (spleen 5–6 cm, liver 3 cm below the costal margin) on physical examination. He had anaemia, eosinophilia (absolute eosinophil count: 1150 mm^–3^), hypogammaglobulinaemia and hypocomplementaemia. Tuberculin skin test and IFN-γ release assays were negative. In lymphocyte subgroup counts, the percentage of B cells was even lower than before (CD19 :  0.8 %) (Table 2).

Chest X-ray showed bilateral extensive infiltrations. Thorax computed tomography (CT) revealed extended nodules with irregular margins and calcifications suggesting granulomatous or infectious aetiology. Hepatosplenomegaly and hypoechoic nodules in spleen, bilateral supraclavicular and axillary lymph nodes reaching 3 cm (US), and retroperitoneal, mesenteric and inguinal microlymphadenopathies (CT) suggested malignancy. Bone marrow aspiration smear and biopsy showed a myeloid leukomoid reaction, supporting an infectious aetiology and excluding haematological malignancy. Lymph node excision biopsy showed non-specific reactive hyperplasia with findings compatible with immunodeficiency, such as regressive lymphoid follicular changes and decreased plasma cells.

He had recurrent infections and multi-system organ involvement such as enteropathy (with associated findings of failure to thrive, anaemia), granulomatous lung disease, nephropathy, non-malignant lymphoproliferation and hypothyroidism. Direct Coombs test positivity supported an autoimmune component of an immunodeficiency syndrome. Additionally, his elder brother (Patient 2), who was known to be healthy on first admission of Patient 1, had been followed with autoimmune lymphoproliferative syndrome (ALPS) in the public hospital for the last year.

Quantitative oxidative burst activity was normal, excluding chronic granulomatous disease. X-linked agammaglobulinaemia was unlikely, although B cell percentage was <1 % because of lymphoproliferation and measurable levels of IgG and IgM. Lymphoproliferation, positive Coombs test and findings of the sibling suggested ALPS but CD4-CD8-TCR α/β [double negative (DN)] T cells were in the normal range (0.39 %). Lymphoproliferation not associated with Epstein–Barr virus (EBV) and negative *SH2D1A* mutation ruled out X-linked lymphoproliferative syndrome. Most clinical and laboratory findings supported CVID as a diagnosis but even so, very early-onset disease and the presence of the other sibling pointed to a monogenic aetiology. Clinical phenotype of the affected two male patients strongly suggested IPEX syndrome but molecular analysis of the *FOXP3* gene was negative.

Prophylaxis with antibiotics and antifungals was started for infection control. Antiviral treatment for cytomegalovirus (CMV) infection was used when necessary. In his 7-year follow-up he had numerous hospitalizations due to respiratory tract infections or findings associated with worsening enteropathy such as hypoalbuminaemia and electrolyte disturbances. Corticosteroids (doses of 0.3–0.5 mg kg^–1^ day^–1^) were used as immunsuppressive agents intermittently at 3–6 month intervals with a slight response. Subcutaneous immunoglobulin replacement therapy was started. He had no matched sibling donor and a matched unrelated donor search was initiated for haematopoietic stem cell transplantation (HSCT).

**Fig. 1.**
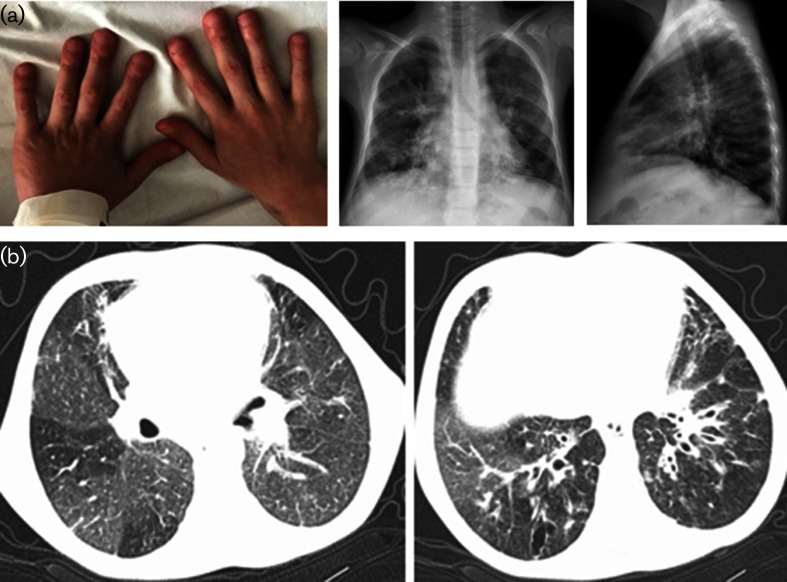
(a) Clubbing and chest X-ray of Patient 1 (at 9 years of age): bilateral heterogeneous ill-defined lung infiltrations with peribronchial thickening. Air trapping on lateral chest X-ray. Mediastinal widening due to lymphadenomegaly. (b) Thorax CT of Patient 1 (at 9 years of age): bilateral mosaic perfusion defect with basal bronchial wall thickening and bronchiectasis.

At the age of 9 years, he had clubbing and chronic lung findings. Chest X-ray showed bilateral extended coarse reticular changes and peribronchial thickenings (Fig. 1a). Thorax CT revealed extended and severe bronchiectasis with thickened bronchial walls, some granulomatous nodules and mosaic appearence, compatible with granulomatous lymphocytic interstitial lung disease (GLILD) (Fig. 1b).

His bone age was 5 years when he was 10 years old. Growth retardation got worse (weight −4.99 SDS, height −3.28 SDS, under 3rd percentile). Vitamin D insufficiency led to hyperparathyroidism, symptomatic hypocalcaemia and severe osteoporosis requiring aggressive calcium and vitamin D replacement [Ca: 5.4 mg dl^−1^, albumin: 2.7 g dl^−1^, parathyroid hormone (PTH): 83.41 pg ml^−1^, vitamin D: 11 ng ml^−1^). Autoantibodies to the thyroid gland (antithyroid peroxidase, antithyroglobulin antibodies) became positive on follow up but thyroid US and thyroid hormones were normal.

### Patient 2

The 7-year-old brother of Patient 1 was hospitalized with cough and respiratory insufficiency. He had recurrent bronchiolitis starting at age 6 months. At the age of 5 years, he had been admitted to the public hospital with dark urination. He had autoimmune haemolytic anaemia (AIHA) (Hb: 5 g dl^−1^, reticulocyte 30 %, direct Coombs +++), hepatosplenomegaly and lymphadenomegalies. He also had proteinuria (10 mg m^–2 ^h^–1^) and low C4 level; kidney biopsy revealed focal segmental glomerulosclerosis. He had been followed for ALPS and prescribed low-dose corticosteroids.

On admission, he had normal growth percentiles (weight 50–75 %, height 25 %), tachypnoea, bilateral rhonchi, fine crackles and hepatosplenomegaly (spleen 2–3 cm, liver 5–6 cm below the costal margin). Complete blood count was normal except a mild lymphopaenia (Table 2). He had mild hypoalbuminaemia (T protein: 6.3 g dl^−1^, albumin: 3 g dl^−1^), high C-reactive protein and erythrocyte sedimentation rate (9.7 mg dl^−1^, 80 mm h^−1^, respectively). He had normal IgG, high IgM and low IgA, IgE and C4 levels (Table 2). Lymphocyte subgroups and CD4-CD8-TCR α/β DN T cells were in the normal range (Table 2). Autoantibodies (antinuclear antibody, antithyroid peroxidase, antithyroglobulin antibodies) were negative while direct Coombs test was highly positive. Thyroid hormones were normal. Tuberculin skin test and IFN-γ release assay were negative. He had no proteinuria. Chest X-ray and thorax CT revealed bilateral extensive infiltrations and findings of chronic bronchitis/bronchiolitis.

He had recurrent/non-persistent diarrhoea (enteropathy), recurrent lower respiratory tract infections, chronic lung disease, a mild nephropathy, non-malign lymphoproliferation, autoimmune haemolytic anaemia, hypoalbuminaemia, some immunoglobulin and complement abnormalities, and a brother with severe enteropathy and lymphoproliferation.

With preliminary diagnosis of IPEX syndrome, prophylaxis with antibiotics and antifungals and regular IVIG replacement were started for infection control. Low-dose corticosteroid (0.3–0.5 mg kg^–1^ day^–1^) was continued for autoimmune cytopaenias.

In his 4-year follow up he had recurrent lower respiratory tract infections and diarrhoea. IgG level declined and IgM level increased progressively. Partial IgA deficiency progressed to complete deficiency and C3/C4 levels normalized over time (Table 2). Subcutaneous immunoglobulin replacement therapy was started. Because he had no matched sibling donor, we began a search for a matched unrelated donor for HSCT.

Haemolytic anaemia attacks generally associated with infections recurred despite oral corticosteroids and mycophenolate mofetil was added. In his 3rd year, he had an abscess in the jaw due to neutropenia (ANC: 447 mm^–3^) and *Staphylococcus aureus* grew in culture. In his 4th year, thrombocytopenia developed requiring high-dose IVIG and pulse methylprednisolone (30 mg kg^–1^ day^–1^) treatments.

Upper and lower gastrointestinal endoscopy revealed bulbitis and duodenitis, and the colon was macroscopically normal. On pathological evaluation, the colon was infiltrated with mixed inflammatory cells including eosinophils. Although he had normal growth percentiles on his first admission, he eventually developed growth retardation (height 3–10 %, weight <3 % at 11 years old).

**Fig. 2.**
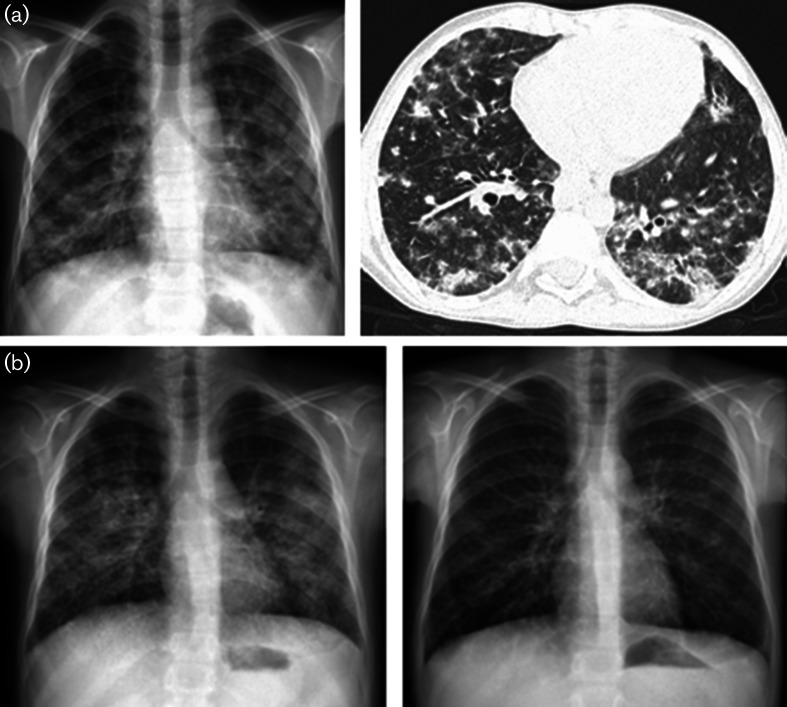
(a) Chest X-ray and thorax CT of Patient 2 (at 10 years of age): bilateral patchy and nodular infiltrations with indistinct margins and coarse septal thickenings. (b) Chest X-ray before and after abatacept therapy (6 months): bilateral hazy parenchymal infiltrations at the start of abatacept therapy. There was almost complete resolution of parenchymal infiltrations with only residual coarse reticular interstitial markings.

He had recurrent lower respiratory tract infections and chronic lung findings. At the age of 10 years, when he presented with respiratory distress, chest X-ray showed patchy infiltrations. Upon thorax CT, bilateral consolidations, inter- and intralobular septal thickenings and mosaic attenuation were found (Fig. 2a). Bronchoscopy revealed hyperaemic, oedematous airways with some yellow–white secretions. Bronchoalveolar lavage fluid was sterile and had a benign cytology. Pathology examination of lung biopsy was compatible with lymphocytic interstitial pneumonia (LIP) and follicular bronchiolitis (there were primary and secondary lymphoid follicle formations scattered in lung parenchyma). Respiratory condition and radiographic findings partially improved with high-dose corticosteroid treatment initiated for immune thrombocytopenia and deteriorated with dose reduction.

**Fig. 3.**
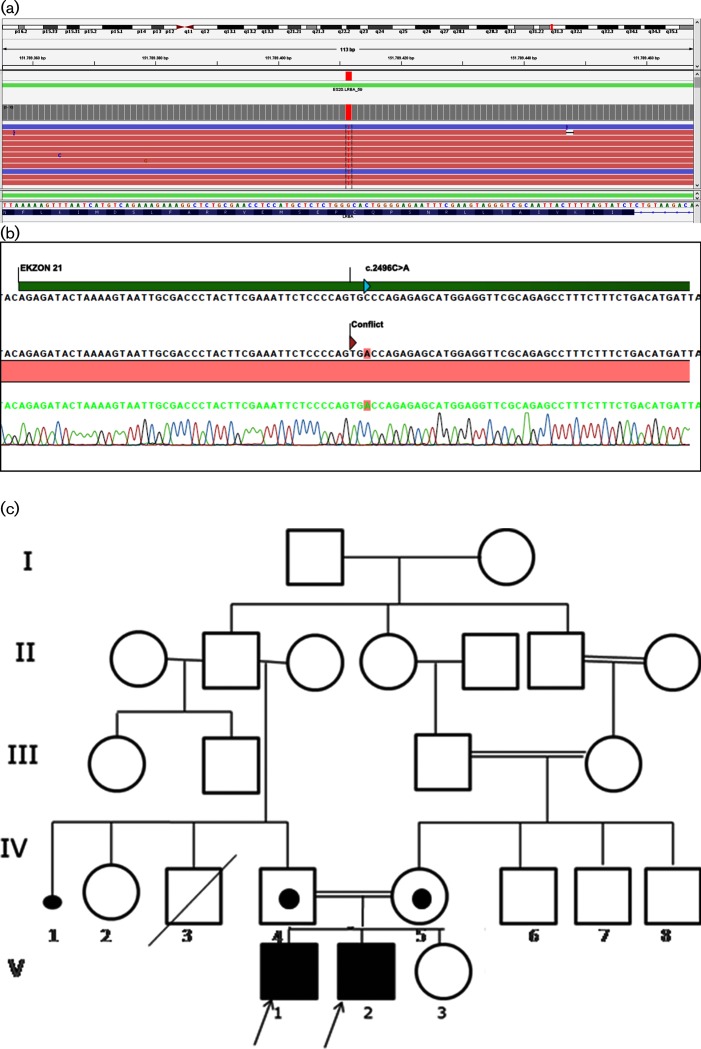
(a) TNGS analysis of the genomic DNA of Patient 1. A nonsense mutation in Exon 21 (c.2496C>A) changes a cystein (C) codon (GCA) into a translation termination (X) codon (TGA) at amino acid position 832 (p.Cys832Ter). (b) Sanger sequencing analysis of the genomic DNA of Patient 1. (c) Pedigree of the family. Affected siblings (V-1 and V-2) are homozygous and both parents (IV-4 and IV-5) are heterozygous for the C832X mutation.

The patients were followed without an exact molecular diagnosis for many years. In 2016, we decided to perform ‘targeted next generation sequencing (TNGS)’ in order to understand the underlying molecular pathology. A TNGS workflow based on an Ion Ampliseq Primary Immune Deficiency Research Panel was designed for sequencing 264 PID genes on an Ion S5 Sequencer. A homozygous c.2496C>A, p.Cys832Ter (p.C832*) mutation was found in the *LRBA* gene in patients 1 and 2 (Fig. 3a). PCR amplification and Sanger sequencing were performed to verify segregation of the mutation in *LRBA* among the family members (Fig. 3b). The mother and father were heterozygous carriers for this mutation as shown on the pedigree of the family (Fig. 3c).

Matched unrelated donors were found for both siblings but their family did not accept HSCT. After molecular diagnosis, abatacept (CTLA-4-Ig fusion protein) was started in both patients (10 mg kg^–1^ per dose, at 2-week intervals, intravenously). Meanwhile, both patients were receiving corticosteroids (0.5 mg kg^–1^ day^–1^) and Patient 2 mycophenolate mofetil as immunosuppressive therapies. Patient 1 had severe oral and oesophageal candidiasis and fungal pneumonia (*Kloeckera apiculata* grew in pharyngeal swab) after the first dose of abatacept leading to cessation of treatment. Mycophenolate mofetil was added for autoimmune enteropathy with some response. Patient 2, after the 6th month of abatacept treatment, showed better clinical condition, with a short hospitalization for a mild lower respiratory tract infection. Chest X ray showed significant improvement (Fig. 2b).

## Discussion

The major clinical findings of the first case were chronic diarrhoea (enteropathy), failure to thrive and nephropathy at admission. He had laboratory abnormalities related to enteropathy (iron deficiency anaemia, hypoalbuminaemia) and immunological defects (low IgA, IgE and C4 levels and B cell percentage). *Salmonella* and CMV infections, positive direct Coombs test, and compensatory hypothyroidism were additional evidence for immune deficiency, autoimmunity and endocrinological involvement, respectively.

During follow-up, Patient 1 developed hepatosplenomegaly, lymphadenomegaly, antithyroid antibody positivity, recurrent pneumonia and granulomatous lung disease. The presence of a brother with autoimmune cytopaenia, lymphoproliferation, recurrent diarrhoea (a milder enteropathy), recurrent respiratory infections, nephropathy and positive direct Coombs test provided evidence of a genetic disease.

The clinical picture of Patient 1 in particular suggested IPEX syndrome. IPEX syndrome is caused by mutations in *FOXP3*, a key transcription factor for Treg cells, causing dysfunction of Treg cells and autoimmunity affecting numerous organs. Autoimmune enteropathy is the leading finding presenting as forms of diarrhoea, gastritis or colitis [10–12]. Patient 1 had chronic diarrhoea starting in the first year of life, resulted in failure to thrive and gastritis and colitis. Patient 2 had a mild, late-onset enteropathy and pathological evidence of gastritis and colitis.

Autoimmune manifestations in IPEX syndrome are generally early-onset findings but may develop later. Autoimmune endocrinopathies [type 1 diabetes mellitus (50 %), thyroiditis (20 %)] and autoimmune cytopenias [AIHA, thrombocytopenia (ITP) and neutropenia] are frequently seen in IPEX patients [10, 11]. Patient 1 had positive antithyroid antibodies and direct Coombs test without cytopenias. Patient 2 showed autoimmune cytopenias in all three series at different ages and even under immune suppressive treatment.

In IPEX syndrome, renal involvement may be seen as tubulonephropathy, nephrotic syndrome, interstitial nephritis and membranous glomerulonephritis [10, 13]. Both patients had proteinuria and renal disease on their first admission.

After many years of follow-up with a clinical diagnosis of IPEX syndrome, it was found that the *FOXP3* gene was not mutated in our patients. The definition IPEX-like disorder had been used for patients having clinical presentations similar to IPEX syndrome, but without mutations in the *FOXP3* gene. Loss-of-function mutations in *CD25* (*IL2RA*), *STAT5b*, *ITCH* and gain-of-function mutations in *STAT1* were found to be responsible for IPEX-like disorders [14, 15].

In IPEX syndrome patients, Treg cells may be in normal numbers with impaired cell function [16, 17]. By contrast, studies investigating Treg cells in IPEX-like disorders revealed different results. Moes *et al*. found that percentages of FOXP3-expressing Treg cells and their suppressive function were normal or reduced in patients who had early-onset autoimmune enteropathy [18]. In another study with IPEX-like patients, Treg cell numbers were reduced but their suppressive function was preserved [14]. These contrasting outcomes may be due to the fact that there are heterogeneous groups of cases without proven molecular defects. While waiting for molecular studies, we followed our patients with the diagnosis of IPEX-like disorders.

Recently, in a boy with IPEX phenotype, an *LRBA* mutation, was detected based on whole exom sequencing. Because of similarity with IPEX syndrome, the investigators examined Treg cells of this patient and five other patients with *LRBA* mutations. CD4+FOXP3+ Treg cells and known cell markers were significantly reduced compared to controls and suppressive function was depressed [19]. Flow cytometric CD4+CD25+FOXP3+ Treg cell percentages were also low in our patients (1.4 and 0.7 % in patients 1 and 2, respectively), but functional studies could not be performed.

Hypogammaglobulinaemia with low or normal B cells is a characteristic feature of CVID patients. Autoimmune, autoinflammatory and lymphoproliferative findings such as enteropathy, GLILD and organomegaly frequently accompany CVID [20]. There are numerous identified genetic defects in CVID phenotype, although some remain to be specified [1]. Many patients having enteropathy and/or GLILD and followed as CVID were finally diagnosed as having CTLA-4 or LRBA deficiency [20], like our patients.

GLILD is frequently seen in patients with LRBA deficiency (38 %), which is known as an immune defect affecting Treg cells [5]. Our first patient had a radiological diagnosis of granulomatous lung disease and bronchiectasis, and the second patient had biopsy-proven intersititial lung disease (LIP and follicular bronchiolitis). Immunohistochemical staining of the specimen revealed a high proliferation index (Ki67), presence of CD20+ B cells and CD3+ T cells within lymphoid aggregates consistent with previous data [21]. Immunohistochemical analysis of specimens from CVID patients diagnosed as GLILD showed a predominance of CD4+ T cells or CD20+ B cells and additionally absence of FOXP3+ Treg cells [22], suggesting that Treg cell dysfunction may contribute to the pathology [20].

A recent consensus statement on the management of GLILD in CVID patients reported corticosteroid use as first-line treatment and azathioprine, rituximab and mycofenolate as second-line agents with more than 80 % agreement [23]. In Patient 2, development of GLILD could not be prevented despite long-term treatment with corticosteroid and mycofenolate mofetil.

The presence of findings of immune dysregulation (in the form of GLILD, enteropathy, AIHA, ITP, diabetes mellitus, neutropenia, hepatitis, arthritis, etc.) may reach 95 % in some cohorts of LRBA-deficient patients. Consequently, corticosteroids and other immunosuppressive or immunomodulatory drugs (such as cyclosporine, mycophenolate mofetil, sirolimus, rituximab, infliximab, rapamycin, hydroxychloroquine, azathioprine and abatacept) are widely used [4–6]. However, most of the reports lack details about response to therapy. In a study including nine patients, three patients with interstitial lung disease were treated with abatacept and showed significant improvement in clinical status, respiratory function and radiological findings. Abatacept, a CTLA-4-immunoglobulin fusion protein, restores the insufficient CTLA-4 function in cells, which is the key point in LRBA deficiency. After the success of that study, they used abatacept in three patients with enteropathy and in a 6-month period two of them showed improvement, one with additional improvement to his uveitis and arthritis [8]. Kostel *et al*. described seven patients with *LRBA* mutations. Systemic corticosteroids, cyclosporine and mycophenolate mofetil were partially effective but could not prevent disease progression and emergence of new autoimmune findings. They used abatacept in two patients: one with GLILD responded well; the other with resistant ITP and enteropathy had no response and died due to intracranial haemorrhage [6].

We started abatacept in our patients after identification of LRBA deficiency. Patient 2 showed good respiratory improvement within  6 months. Patient 1 developed fungal oesophagitis and pneumonia in a few days after the first dose of abatacept, leading to interruption of treatment, and responded well to antifungal treatment. Fungal infections probably related to immunosuppressive therapy were reported in LRBA-deficient patients [5, 6]. Two patients under abatacept therapy were reported as acquiring chronic norovirus infection, but this was also defined in CVID and LRBA patients who did not take any medication [8]. Patient 1 responded partially to mycofenolate mofetil in terms of enteropathy and hospital admissions. In the context of a lack of a clear relationship between fungal infection and medication, we decided to start abatacept again.

Given the different outcomes associated with immunosuppressive therapies, HSCT seems to be the only curative treatment in LRBA patients. Eight of 12 patients treated with HSCT survived (67 %) as of this writing, most of them with good response [24]. We planned HSCT for our patients long before the genetic diagnosis of LRBA deficiency. Although donors were found recently for both of them, the rejection of HSCT by the family makes immunosuppressive treatment the only option for now.

## Conclusion

Recurrent infections, autoimmunity affecting multiple organ systems, lymphoproliferation and hypo/dysgammaglobulinaemias should be important suggestive findings for immune dysregulation syndromes. It should be kept in mind that both similar clinical presentations of different genetic defects and different findings of the same mutation are possible.

Early identification of the disease and early intervention with HSCT may be life saving in LRBA deficiency as with many other immunodeficiency syndromes. Immunosuppressive agents are good treatment options in the period between diagnosis and HSCT.
